# Sodium–Glucose Cotransporter 2 Inhibitors as Potential Antioxidant Therapeutic Agents in Cardiovascular and Renal Diseases

**DOI:** 10.3390/antiox14030336

**Published:** 2025-03-13

**Authors:** Tapan A. Patel, Hong Zheng, Kaushik P. Patel

**Affiliations:** 1 Department of Cellular and Integrative Physiology, University of Nebraska Medical Center (UNMC), Omaha, NE 68198, USA; kpatel@unmc.edu; 2 Division of Basic Biomedical Sciences, Sanford School of Medicine of the University of South Dakota, Vermillion, SD 57069, USA

**Keywords:** SGLT2 inhibitors, reactive oxygen species (ROS), free radicals, oxidative stress (OS), cardio-renal diseases

## Abstract

Redox (reduction–oxidation) imbalance is a physiological feature regulated by a well-maintained equilibrium between reactive oxygen species (ROS) and oxidative stress (OS), the defense system of the body (antioxidant enzymes). The redox system comprises regulated levels of ROS in the cells, tissues and the overall organ system. The levels of ROS are synchronized by gradients of electrons that are generated due to sequential reduction and oxidation of various biomolecules by various enzymes. Such redox reactions are present in each cell, irrespective of any tissue or organ. Failure in such coordinated regulation of redox reactions leads to the production of excessive ROS and free radicals. Excessively produced free radicals and oxidative stress affect various cellular and molecular processes required for cell survival and growth, leading to pathophysiological conditions and, ultimately, organ failure. Overproduction of free radicals and oxidative stress are the key factors involved in the onset and progression of pathophysiological conditions associated with various cardiovascular and renal diseases. Sodium–glucose cotransporter 2 inhibitors (SGLT2is) are glucose-lowering drugs prescribed to diabetic patients. Interestingly, apart from their glucose-lowering effect, these drugs exhibit beneficial effects in non-diabetic patients suffering from various cardiovascular and chronic kidney diseases, perhaps due to their antioxidant properties. Recently, it has been demonstrated that SGLT2is exhibit strong antioxidant properties by reducing ROS and OS. Hence, in this review, we aim to present the novel antioxidant role of SGLT2is and their consequent beneficial effects in various cardiovascular and renal disease states.

## 1. Introduction

Redox imbalance and augmented oxidative stress (OS) indicate disequilibrium between the generation of free radicals or reactive oxygen species (ROS) and cellular defense or the antioxidant system [1,2,3]. ROS include various free radicals, such as hydroxyl radical (OH·) and superoxide (O_2_·^−^), as well as different non-radical species, such as hydrogen peroxide (H_2_O_2_) and peroxynitrite (ONOO^−^) [4,5]. Various biochemical reactions in the cell result in the constant production of ROS. During redox imbalance and OS, there is an enhanced level of ROS, as well as reactive nitrogen species (RNS). Such enhanced ROS and RNS oxidize various biomolecules, like proteins, lipids and DNA (deoxyribonucleic acid), present in the cell and cell organelles, leading to cellular dysfunction, tissue deterioration and cell death [6,7,8,9,10]. Increased OS, ROS and RNS have been associated with various cardiovascular diseases, such as congestive heart failure (CHF), hypertension, cardiac arrhythmias, coronary heart disease, atherosclerotic plaque formation, etc. [11,12,13,14,15,16,17]. Significantly increased ROS levels were documented in the Peripheral Blood Mononuclear Cell (PBMC) mitochondria of CHF patients. These increased levels of ROS were positively associated with urinary 8-hydroxydeoxyguanosine (a systemic oxidative stress marker), as well as disease severity in CHF patients [18]. Furthermore, increased ROS and abnormal calcium release via alterations in type 2 ryanodine receptor (RyR2) promote atrial fibrillation (AF). Inhibition of ROS production and RyR2 leakage exhibits a preventive effect against AF [19]. Also, increased ROS levels lead to oxidation of proteins, resulting in protein carbonylation (PCO). Such carbonylated proteins have been found to be significantly increased in the myocardium of patients with HF [20]. Moreover, enhanced ROS production by nicotinamide adenine dinucleotide phosphate (NADPH) oxidase and increased NADPH oxidase activity have been documented in failing human hearts [21,22]. Upregulation of NADPH oxidase subunits was also observed in the hearts of diabetic rats, indicating increased OS [23]. Also, a significant increase in the production of ROS and remarkable upregulation of NADPH oxidase subunits was observed in the left ventricle (LV) of rats with CHF [24], which may be partly attributed to the enhanced sympathetic tone observed in the rats with CHF. Recently, it was demonstrated that human microvascular endothelial cells (HMEC-1) showed upregulation of the NADPH oxidase subunits p22phox and Nox4, as well as sodium–glucose cotransporter 2 (SGLT2), after they were exposed to the plasma of patients with cardiogenic shock and isoproterenol (a non-selective beta-adrenergic receptor agonist) [25], indicating a direct link between enhanced sympathetic tone and increased OS. Such an enhanced sympathetic tone is the key characteristic of CHF, as demonstrated by increased Norepinephrine turnover in the left ventricles, urine, serum and kidneys of rats with CHF [26,27,28]. Another lethal consequence of increased OS is lipid peroxidation (LPO), which is the metabolic process of the oxidation of lipids by ROS, which leads to the initiation of apoptosis and autophagy [29]. Increased lipid peroxides and malondialdehyde (a highly toxic compound generated from lipid oxidation) have been noted in the plasma of patients with CHF [30]. Increased ROS levels affect vascular function via oxidative damage by generating free radicals, reducing the synthesis of nitric oxide (NO) and potentially leading to hypertension [17,31].

**Figure 1:**
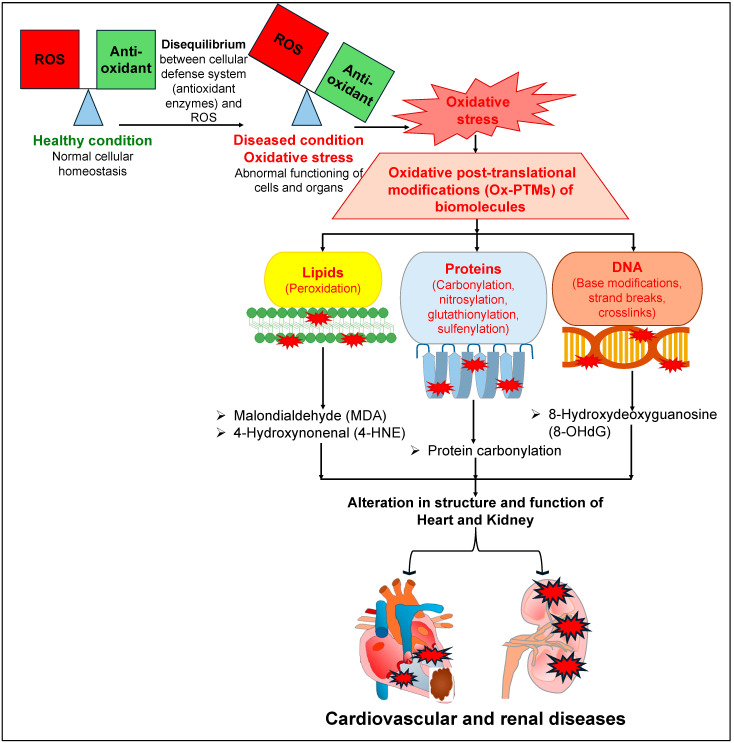
A detailed mechanism elucidating the deleterious effects of oxidative stress on different biomolecules, leading to different cardiovascular and renal diseases.

Similarly to the deleterious roles of OS, ROS and RNS in the pathophysiology of cardiovascular diseases (CVDs), they also affect normal renal physiology, leading to the development and progression of kidney diseases [32,33,34]. Increased ROS levels and redox imbalance in the kidney affect renal tubular transport, leading to disruptions to the regulation of various solutes and water reabsorption in the kidney [35,36,37], which is a key process required to maintain a balance between the amount of electrolytes and the volume of extracellular fluid in the body. Several reports have documented that increased OS damages the renal tubular cells, leading to cell death and renal injury [38,39,40,41]. Further, increased activity of NADPH oxidase or its expression leads to a rise in the levels of ROS, and such augmented ROS levels have been reported in patients, as well as in animal models, with compromised renal function [39,42,43,44]. There was a remarkable reduction in the levels of CuZn superoxide dismutase (SOD) and Mn SOD (key enzymes involved in the regulation of superoxide radicals), but an increased level of gp91phox (a subunit of NADPH oxidase involved in the production of superoxide anion), in the kidneys of rats with chronic renal failure (5/6 nephrectomized) [45]. Consistently with these observations, there were significantly elevated levels of carbonylated proteins and oxidized human serum albumin (HSA) in the plasma of patients with chronic kidney disease (CKD) [46]. Intriguingly, several reports document that patients with heart failure also suffer from CKD [47,48,49]. Conversely, patients suffering from CKD have a higher risk of CVDs as well [50,51,52]. All of these reports indicate that patients with CKD or heart failure exhibit high comorbidity. The augmented ROS and OS levels that have been characteristically observed in both CVDs and CKD may be one of the common key links for this comorbidity. Furthermore, augmented levels of ROS lead to OS, which in turn leads to various types of oxidative post-translational modifications (Ox-PTMs) of biomolecules such as lipids (peroxidation), proteins (carbonylation, nitrosylation, glutathionylation, sulfenylation) and DNA (base modifications, strand breaks, crosslinks) [10,53,54]. Such elevated levels of Ox-PTMs may affect the structure and function of various organs, including the heart and the kidneys (Figure 1). Such augmented ROS and free radicals are neutralized by two mechanisms: either by antioxidant enzymes, such as SOD, catalase (CAT), and glutathione peroxidase (GPx), or by non-enzymatic antioxidant biomolecules, such as flavonoids, vitamin C, and vitamin E [55]. Hence, they are routinely recommended as a preventive measure against OS-associated diseases, including CVDs and renal diseases, to help maintain a healthy outcome [55,56,57,58,59,60].

SGLT2 inhibitors were developed primarily to treat type 2 diabetes mellitus by reducing blood glucose levels [61,62]. SGLT2 inhibitors exhibit cardio-renal protection in both diabetic and non-diabetic patients [63,64,65,66], by normalizing various metabolic (inhibiting glucose reabsorption in the renal proximal tubular cells and enhancing urinary glucose excretion) and hemodynamic effects (regulating blood pressure). The contribution of SGLT2 inhibitors to cardio-renal protection includes various mechanisms, such as natriuresis, reducing blood pressure, lowering blood glucose levels, improving renal and vascular function, altering the molecular signaling pathways related to energy metabolism and endothelial function, inducing a metabolic shift in substrate utilization from carbohydrate to lipids or ketone bodies (gluconeogenesis and ketosis) as the metabolic substrate, and reduced oxidative stress [67,68,69,70,71]. All of these mechanisms indicate the biochemical regulatory interactions between the heart and kidneys, which are thought to exhibit protective outcomes in different cardiovascular and renal diseases. Chronic hyperglycemia (a diabetic condition) leads to a decrease in insulin secretion and an increase in insulin resistance, which causes glucotoxicity and oxidative stress [72,73]. Diabetic patients are at risk of various cardiovascular and renal diseases [74,75]. Interestingly, SGLT2 inhibitors can reduce glucotoxicity in renal tubular cells by reducing glucose reabsorption and mitochondrial dysfunction, reducing renal hypoxia by reducing oxygen demand and improving beta cell function [76,77,78]. A meta-analysis of randomized controlled trials revealed that treatment of heart failure patients with SGLT2 inhibitors showed a remarkable reduction in hospitalization and cardiovascular death. In these patients, treatment with SGLT2 inhibitors improved metabolic indices, and reduced body weight, blood pressure and plasma glucose levels [79,80,81].

Different SGLT2is approved by the Food and Drug Administration (FDA) include canagliflozin (Invokana), dapagliflozin (Farxiga), empagliflozin (Jardiance), ertugliflozin (Steglatro) and bexagliflozin (Brenzavvy) [70,82]. Intriguingly, several reports have documented that, clinically, these SGLT2is prove to be beneficial for patients suffering from CVDs, reducing the risk of death and hospitalization [83,84]. SGLT2is have also showed beneficial effects in patients suffering from renal diseases, such as CKD in diabetes, reducing their pathophysiology [85,86,87]. SGLT2is exhibit common pharmacokinetic characteristics, such as fast oral absorption, long elimination half-lives (allows for the administration of one dose daily), high plasma protein binding (PPB) capacity, wide tissue distribution and extensive biotransformation by glucuronidation [88,89,90,91]. Because of such pharmacokinetic characteristics, SGLT2is might show antioxidant effects and cardio-renal protection by reducing either ROS or glucotoxicity. The clinical significance of such a direct link is unclear, and remains to be examined.

However, the exact molecular mechanism of the beneficial effect of these SGLT2is is unknown in CVDs, as well as in renal diseases such as CKD. Hence, this review aims to explore the evidence for the role of SGLT2is as potential antioxidant agents in various CVDs and renal diseases.

## 2. SGLT2 Inhibitors as Antioxidants in Cardiovascular Diseases

SGLT2is may prevent cardiovascular diseases by several mechanisms, such as by activating the cellular defense (antioxidant) system, by scavenging ROS or by reducing ROS-producing enzymes.

### 2.1. Empagliflozin (EMPA) in Cardiovascular Diseases

A recent study reported that EMPA reduced cardiomyocyte hypertrophy and myocardial OS by suppressing advanced oxidation protein product (AOPP) levels and reducing the expression of NADPH oxidase 2 (NOX2) in rats with myocardial infarction (MI) [92], indicating a reduction in myocardial OS. The primary function of NOXs, a family of electron-transporting membrane enzymes, is the production of ROS, which are the key contributors to oxidative damage under different pathologic disease conditions [93]. Furthermore, EMPA treatment produced a significant decrease in the levels of H_2_O_2_, lipid peroxides and 3-nitrotyrosine (oxidative parameters) in the left ventricular (LV) myocardium of patients with heart failure with preserved ejection fraction (HFpEF), as well as in Zucker diabetic fatty (ZDF) obese rats (dubbed as a model of HFpEF) [94]. Interestingly, a significant improvement in diastolic function was observed and confirmed by invasive hemodynamics in a non-diabetic pig model of HF after EMPA treatment. Also, EMPA treatment improved NO signaling (endothelial nitric oxide synthetase-eNOS). In the same pig model, myocardial OS was also reduced by EMPA via reduction of 8-hydroxydeoxyguanosine (a marker of nuclear OS) and malondialdehyde (a marker of lipid OS), and also via improving the activity of SOD (the antioxidant enzyme) [95], indicating that EMPA is a powerful antioxidant. Likewise, EMPA administration to mice with diabetic cardiomyopathy (DCM) showed a significant reduction in myocardial OS injury by reducing the levels of OS markers (lipid hydroperoxide and malondialdehyde (MDA)) and increasing the levels of SOD and GSH-Px in the cardiac tissue [96].

### 2.2. Dapagliflozin (DAPA) in Cardiovascular Diseases

A significant reduction in OS in aortic vessels and improved endothelial function in diabetic rats after DAPA treatment have been reported [97]. In vivo and in vitro studies showed that DAPA treatment improved cardiac dysfunction and ameliorated DCM by reducing high glucose (HG)-induced OS. DAPA treatment attenuated HG-induced cell death and ROS levels in rat embryonic cardiac myoblasts (H9C2 cells). Also, DAPA treatment significantly reduced myocardial NOX subunits (gp91phox and p22phox) in rats with streptozotocin (STZ)-induced DCM, as well as reducing NOX subunits and the production of H_2_O_2_ in HG-treated H9C2 cells [98], exhibiting a prophylactic role in ROS production. Both EMPA and DAPA treatment showed a significant reduction in Tumor Necrosis Factor-α (TNF-α)-induced intracellular ROS levels and improved NO levels in human endothelial cells [99]. In endothelial cells, DAPA treatment showed a significant reduction in H_2_O_2_-induced intracellular ROS and ONOO^–^, as well as an improvement in the H_2_O_2_-induced decline in NO levels [100], which indicates that DAPA improves endothelial dysfunction by reducing ROS and improving NO levels. An in vivo study using pulmonary hypertension (PH)-induced right heart failure (RHF) rat models indicated that DAPA improved PH-induced anatomical and functional alterations in the right heart by reducing the ROS levels [101]. The myocardial OS inhibitory activity of DAPA was observed in mice with MI. In this model, DAPA caused a remarkable reduction in ROS production via reducing NOX2 and NOX4, and also significantly improved the activity of SOD and enhanced the levels of nuclear factor erythroid 2-related factor 2 (NRF2) and NADPH quinone dehydrogenase 1 (NQO1) [102]. NRF2 and NQO1 are key proteins involved in the cellular antioxidant defense system. Pretreatment of cardiac fibroblasts (CFs) with EMPA and DAPA provided a remarkable reduction in Ang II/TGF-β1-induced augmented levels of ROS and MDA (a key biomarker for OS from LPO) content. Also, EMPA and DAPA exhibited a marked reduction in Ang II/TGF-β1-induced upregulation of NOX4 protein in CFs [103].

### 2.3. Canagliflozin (CANA) in Cardiovascular Diseases

Recently, it was shown that CANA treatment significantly reduced Palmitic Acid (PA)-induced intracellular ROS levels, lipid ROS levels and cell aging in vascular endothelial cells [104]. Furthermore, EMPA and DAPA treatment of human atrial tissue samples from non-diabetic patients with HF provided a significant reduction in ROS levels and OS, as well as reducing the expression of monoamine oxidase-A (MAO-A) and MAO-B [105]. These monoamine oxidases (MAOs) are key sources of ROS and they augment OS [106,107]. CANA administration mitigated heart dysfunction in STZ-induced diabetic mice, as well as causing a marked reduction in PA-induced ROS levels and lipotoxicity in mouse cardiomyocytes (HL-1 cells) [108]. A DCM mice model showed that CANA treatment prevented myocardial injury and significantly reduced the contents of MDA and PCO. Additionally, CANA also improved the activities of SOD and CAT. Furthermore, CANA treatment reduced the augmented ROS and PCO levels, in addition to improving the activities of SOD and CAT, in HG-treated H9C2 cells [109], representing an antioxidative role against myocardial OS and injury. Ex vivo treatment of human atrial myocardial tissue from diabetic patients with CANA caused a remarkable reduction in superoxide production, the activity of NOX and OS, and improved NOS coupling. Similarly, CANA treatment prevented the generation of superoxide and the activity of NOX in primary human cardiomyocytes (hCMs) [110]. In a canine (beagle dogs) atrial fibrillation (AF) model, oral administration of CANA provided a marked reduction in the enhanced levels of ROS in the left atrial cardiomyocytes [111]. In a chronic myocardial ischemia Yorkshire swine model, oral administration of CANA caused a remarkable decline in OS via significantly reducing total protein oxidation in the myocardium, with concomitant upregulation of mitochondrial SOD2 and improvement of hemodynamic parameters [112], indicating an improvement in overall myocardial function. Taken together, these data show that SGTL2is have a significant role in reducing OS and improving cardiac function. They exhibit a potential antioxidative role against various CVDs.

## 3. SGLT2 Inhibitors as Antioxidants in Renal Diseases

Several clinical trials and meta-analysis of SGLT2 inhibitor trials have reported that SGLT2is reduced the risk of advancement of renal diseases or death due to cardiovascular problems in patients with CKDs [113,114,115]. This renoprotective role of SGLT2is may be due to their antioxidative properties.

### 3.1. Empagliflozin (EMPA) in Renal Diseases

Previous in vitro studies on human proximal tubular cells (PTCs) reported that EMPA decreased HG-induced cell death and enhanced the functions of mitochondria by normalizing ROS production (including superoxide and hydrogen peroxide), adenosine triphosphate (ATP) generation, mitochondrial membrane potential (MMP), LPO and MDA levels [116,117,118]. In an in vivo study using a mouse model with diabetic kidney disease (DKD), EMPA administration resulted in a marked reduction in tubular injury and ROS levels, as well as upregulation of GPx4 (antioxidant enzyme) [117], indicating an improved equilibrium between ROS and the antioxidant system. In addition, EMPA showed anti-inflammatory and antioxidant effects against nephropathy in an STZ-induced diabetic rat model. These effects were demonstrated by a decline in renal MDA, TNF-α and MCP-1 levels, and elevated activities of renal SOD and GPx [119]. Interestingly, spontaneously hypertensive rats (SHRs) expressing human C-reactive protein (CRP) (SHR-CRP) showed a significant increase in OS in the renal cortex. In this animal model, EMPA treatment mitigated OS by reducing the LPO and increasing the activities of antioxidant enzymes (GPx, CAT) and the levels of reduced glutathione (GSH—a non-enzymatic antioxidant) [120]. In an LLC-PK1 model of diabetic nephropathy (by exposing cells to HG) and OS (by exposing cells to H_2_O_2_), EMPA treatment showed improvements in cell viability and GSH levels [121]. Furthermore, in an in vivo and in vitro Tacrolimus-induced renal injury model, EMPA treatment also showed a significant reduction in ROS production and improved cell viability [122]. Recent clinical data show that treatment of diabetic nephropathy (DN) patients with SGLT2is (DAPA, EMPA or CANA) for 12 weeks significantly reduced urinary 8-hydroxydeoxyguanosine (8-OHdG) levels (a marker of OS) and proteinuria [123], exhibiting an antioxidative role in improving renal function in nephropathy.

### 3.2. Dapagliflozin (DAPA) in Renal Diseases

Recently, DAPA treatment was shown to cause remarkable reductions in palmitate-induced proximal tubular cell injury, mitochondrial dysfunction and OS [124]. A high-fat diet (HFD)-induced renal injury model exhibited marked reduction in renal injury after 4 weeks of DAPA treatment, via diminishing of the renal OS and improvements in renal function [125,126]. In an in vitro H_2_O_2_-induced human proximal tubular cell injury model (using HK-2 cells), with augmented cytosolic and mitochondrial ROS, DAPA treatment showed a remarkable reduction in ROS production and cell death [127], exerting a protective role against OS and OS-induced cellular damage. In addition to reducing ROS, DAPA treatment also provided improvements in mitochondrial integrity, the expression of the respiratory chain complex and the number of mitochondria in an adenine-induced renal injury model. In the same animal model, DAPA also improved the activity of SOD and levels of GSH, whereas it reduced levels of MDA in the kidneys [128]. Recently, in a STZ-induced diabetic nephropathy (DN) rat model, DAPA treatment showed marked protective action against renal damage, apoptosis and OS. DAPA protects this renal damage by reducing the expression of kidney injury molecule 1 (Kim1) and Nephrin (Nphs1-a key biomarker of glomerular injury). The anti-apoptotic activity of DAPA against DN was exerted by reducing the expression of renal Bax (pro-apoptotic protein) and the ratio of Bax/Blc2. The OS-reducing activity of DAPA, in an STZ-induced DN rat model, was represented by declining levels of gp91phox (a subunit of NOX) and improvements in the levels of MnSOD [129]. Interestingly, in a DN model of db/db mice, the renal cortex showed augmented levels of ROS, upregulation of NOX4 and increased gene expression of caspase-12 and Bax (the proapoptotic factors), which were attenuated by long-term treatment (12 weeks) with DAPA [130]. Furthermore, six weeks of oral administration of DAPA to high-salt (HS) diet-fed Dahl salt-sensitive rats showed downregulation of NOX2, NOX4 and free radical generation, and upregulation of antioxidant enzymes (SOD and CAT) [131], indicating the renoprotective action of DAPA in a non-diabetic model of cardio-renal syndrome.

### 3.3. Canagliflozin (CANA) in Renal Diseases

**Figure 2:**
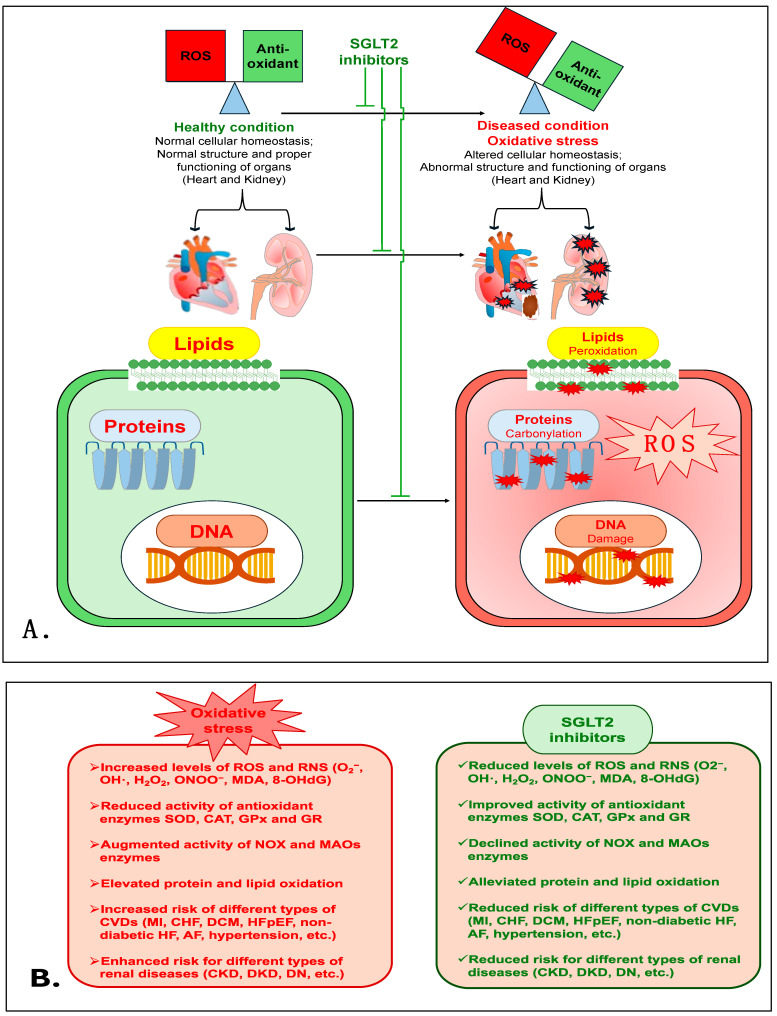
(**A**). The proposed mechanism elucidates the antioxidative role of SGLT2is in cardiovascular and renal diseases. (**B**). The detrimental effects of oxidative stress and the protective mechanism of SGLT2is.

Treatment with another SGLT2i, CANA, was shown to cause a marked reduction in isoprenaline (ISO)-induced renal oxidative damage in a rat model that mimicked enhanced sympathetic nervous system (SNS)-induced injury. In this rat model, CANA treatment prevented the enhancement of OS markers (MDA, myeloperoxidase (MPO) and advanced protein oxidation product (APOP)). CANA also reduced NOX4 levels and improved the activity of antioxidant enzymes (CAT and SOD) and levels of GSH [132]. Interestingly, in type 2 diabetes mellitus (Otsuka Long-Evans Tokushima Fatty-OLETF) rats, CANA treatment was shown to have a renoprotective action via reducing lipid peroxides (MDA + 4HNE) and NOX proteins (NOX2 and NOX4) in the kidneys when combined with fasting before myocardial infarction (MI) [133]. This study indicates the potential antioxidant role of CANA in the renal tissue of diabetic rats with type 1 cardio-renal syndrome. Additionally, CANA treatment mitigated adenine-induced CKD in rats, and improved antioxidant enzymes such as SOD, CAT and glutathione reductase (GR) and total antioxidant activity in renal tissue homogenate [134]. CANA treatment also prevented HG-induced apoptosis and OS in normal rat renal tubular epithelial cells (NRK-52E) via suppressing ROS production. CANA also significantly improved the activity of SOD, CAT and GPx and the expression of Nrf2, whereas it markedly reduced MDA levels and the expression of NOX4, indicating that OS diminishes the role of CANA [135]. Taken together, these data demonstrate that SGLT2is are powerful antioxidants, and have a strong capacity to reduce OS and prevent renal damage and its associated pathophysiology. Figure 2 is a schematic of the various mechanisms that are involved in the antioxidative role of SGLT2is in cardiovascular and renal diseases. Table 1 shows the antioxidative effects of various SGLT2is in numerous cardiovascular and renal disease models in a number of different studies.

## 4. Conclusions and Future Perspectives

Enhanced oxidative stress and ROS are major contributors to the pathophysiology of various cardiovascular and renal diseases. They affect the physiology and function of the heart and kidneys. Various factors may contribute to the enhancement of oxidative stress, such as alterations in the activity of antioxidant enzymes (SOD, CAT, GPx and GR), augmentations in the activity of NOX enzymes, etc. Our review summarizes the scientific evidence that SGLT2is exhibit a remarkable antioxidative role against oxidative stress and prevent deleterious effects on the heart and kidneys. This review presents SGLT2is as potent antioxidative therapeutic agents in diverse cardiovascular and renal diseases. Apart from diabetes, SGLT2is are beneficial in cardiovascular and renal diseases, which may be partly attributed to their capability to reduce oxidative stress and ROS in the heart and kidneys. Hence, further detailed studies (including preclinical and clinical studies) in different animal models are required to validate the antioxidant properties and beneficial effects that SGLT2is provide via reducing ROS and preventing Ox-PTMs, as well as to explore the underlying molecular mechanisms of SGLT2is in various cardiovascular and renal disease conditions.

## Figures and Tables

**Table 1:** Antioxidant roles of various SGLT2 inhibitors (SGLT2is) in different cardiovascular and renal diseases.

SGLT2 Inhibitor	Disease Model/Pathology	Species	Mechanism of Action	References
Empagliflozin	Myocardial infarction (MI)	Sprague–Dawley rats	Lowering AOPP levels and expression of NOX2	[92]
Empagliflozin	Heart failure with preserved ejection fraction (HFpEF)	ZDF obese rats	Attenuating levels of H_2_O_2_, lipid peroxides and 3-nitrotyrosine	[94]
Empagliflozin	Non-diabetic heart failure	Yorkshire swine	Reducing levels of 8-OHdG and MDA and enhancing activity of SOD	[95]
Empagliflozin	Diabetic cardiomyopathy (DCM)	KK-Ay mice	Causing decline in lipid hydroperoxide and MDA levels, as well as augmenting levels of SOD and GPx	[96]
Dapagliflozin	Diabetic cardiomyopathy (DCM)	Sprague–Dawley rats	Reducing myocardial NOX subunits (gp91phox and p22phox)	[98]
Dapagliflozin	Pulmonary hypertension (PH)-induced right heart failure (RHF)	Sprague–Dawley rats	Cause decline in ROS levels	[101]
Dapagliflozin	Myocardial infarction (MI)	C57BL/6 mice	Reducing levels of NOX2 and NOX4, together with enhancing SOD, NRF2 and NQO1	[102]
Canagliflozin	Diabetic cardiomyopathy (DCM)	C57BL/6J mice	Cause decline in levels of MDA and PCO, along with improving activities of SOD and CAT	[109]
Canagliflozin	Atrial fibrillation (AF) model	Beagle dogs	Reducing levels of ROS	[111]
Canagliflozin	Chronic myocardial ischemia	Yorkshire swine	Decreasing total protein oxidation and upregulating mitochondrial SOD2	[112]
Empagliflozin	Diabetic kidney disease (DKD)	C57BL/6 mice	Reducing ROS levels and upregulating GPx4	[117]
Empagliflozin	STZ-induced diabetic nephropathy (DN)	Wistar rats	Declining MDA levels and enhancing activities of SOD and GPx	[119]
Empagliflozin	Renal injury model	Sprague–Dawley rats	Reducing 8-OHdG levels	[122]
Dapagliflozin	High-fat diet (HFD)-induced renal injury model	Wistar rats	Lowering MDA and 4-hydroxynonenal (4-HNE) levels; reducing expression of NOX4 and renal OS	[125,126]
Dapagliflozin	Adenine-induced renal injury model	C57BL/6J mice	Reducing levels of MDA, and improving activity of SOD and levels of GSH	[128]
Dapagliflozin	STZ-induced DN	Wistar rats	Lowering levels of gp91phox and improving levels of MnSOD	[129]
Canagliflozin	Isoprenaline (ISO)-induced renal oxidative stress model	Long Evans rats	Decreasing levels of MDA, MPO, APOP and NOX4, and enhancing activity of CAT and SOD and levels of GSH	[132]
Canagliflozin	Adenine-induced chronic kidney disease (CKD)	Wistar rats	Improving activities of SOD, CAT and glutathione reductase (GR) and total antioxidant activity	[134]

## References

[1] Jena A.B., Samal R.R., Bhol N.K., Duttaroy A.K. (2023). Cellular Red-Ox system in health and disease: The latest update. Biomed. Pharmacother..

[2] Afzal S., Abdul Manap A.S., Attiq A., Albokhadaim I., Kandeel M., Alhojaily S.M. (2023). From imbalance to impairment: The central role of reactive oxygen species in oxidative stress-induced disorders and therapeutic exploration. Front. Pharmacol..

[3] Betteridge D.J. (2000). What is oxidative stress?. Metabolism.

[4] Rauf A., Khalil A.A., Awadallah S., Khan S.A., Abu-Izneid T., Kamran M., Hemeg H.A., Mubarak M.S., Khalid A., Wilairatana P. (2024). Reactive oxygen species in biological systems: Pathways, associated diseases, and potential inhibitors—A review. Food Sci. Nutr..

[5] Zhang J., Wang X., Vikash V., Ye Q., Wu D., Liu Y., Dong W. (2016). ROS and ROS-Mediated Cellular Signaling. Oxid. Med. Cell Longev..

[6] Pacher P., Beckman J.S., Liaudet L. (2007). Nitric oxide and peroxynitrite in health and disease. Physiol. Rev..

[7] Trachootham D., Lu W., Ogasawara M.A., Nilsa R.D., Huang P. (2008). Redox regulation of cell survival. Antioxid. Redox Signal.

[8] Cecarini V., Gee J., Fioretti E., Amici M., Angeletti M., Eleuteri A.M., Keller J.N. (2007). Protein oxidation and cellular homeostasis: Emphasis on metabolism. Biochim. Biophys. Acta.

[9] Ryter S.W., Kim H.P., Hoetzel A., Park J.W., Nakahira K., Wang X., Choi A.M. (2007). Mechanisms of cell death in oxidative stress. Antioxid. Redox Signal.

[10] Juan C.A., de la Lastra J.M.P., Plou F.J., Pérez-Lebeña E. (2021). The Chemistry of Reactive Oxygen Species (ROS) Revisited: Outlining Their Role in Biological Macromolecules (DNA, Lipids and Proteins) and Induced Pathologies. Int. J. Mol. Sci..

[11] Panda P., Verma H.K., Lakkakula S., Merchant N., Kadir F., Rahman S., Jeffree M.S., Lakkakula B., Rao P.V. (2022). Biomarkers of Oxidative Stress Tethered to Cardiovascular Diseases. Oxid. Med. Cell Longev..

[12] Afanas’ev I. (2011). ROS and RNS signaling in heart disorders: Could antioxidant treatment be successful?. Oxid. Med. Cell Longev..

[13] Leopold J.A., Loscalzo J. (2008). Oxidative mechanisms and atherothrombotic cardiovascular disease. Drug Discov. Today Ther. Strateg..

[14] Nowak W.N., Deng J., Ruan X.Z., Xu Q. (2017). Reactive Oxygen Species Generation and Atherosclerosis. Arterioscler. Thromb. Vasc. Biol..

[15] Jeong E.M., Liu M., Sturdy M., Gao G., Varghese S.T., Sovari A.A., Dudley S.C. (2012). Metabolic stress, reactive oxygen species, and arrhythmia. J. Mol. Cell Cardiol..

[16] Sugamura K., Keaney J.F. (2011). Reactive oxygen species in cardiovascular disease. Free Radic. Biol. Med..

[17] Rodrigo R., Gonzalez J., Paoletto F. (2011). The role of oxidative stress in the pathophysiology of hypertension. Hypertens. Res..

[18] Shirakawa R., Yokota T., Nakajima T., Takada S., Yamane M., Furihata T., Maekawa S., Nambu H., Katayama T., Fukushima A. (2019). Mitochondrial reactive oxygen species generation in blood cells is associated with disease severity and exercise intolerance in heart failure patients. Sci. Rep..

[19] Xie W., Santulli G., Reiken S.R., Yuan Q., Osborne B.W., Chen B.X., Marks A.R. (2015). Mitochondrial oxidative stress promotes atrial fibrillation. Sci. Rep..

[20] Brioschi M., Polvani G., Fratto P., Parolari A., Agostoni P., Tremoli E., Banfi C. (2012). Redox proteomics identification of oxidatively modified myocardial proteins in human heart failure: Implications for protein function. PLoS ONE.

[21] Borchi E., Bargelli V., Stillitano F., Giordano C., Sebastiani M., Nassi P.A., d’Amati G., Cerbai E., Nediani C. (2010). Enhanced ROS production by NADPH oxidase is correlated to changes in antioxidant enzyme activity in human heart failure. Biochim. Biophys. Acta.

[22] Heymes C., Bendall J.K., Ratajczak P., Cave A.C., Samuel J.L., Hasenfuss G., Shah A.M. (2003). Increased myocardial NADPH oxidase activity in human heart failure. J. Am. Coll. Cardiol..

[23] Sharma N.M., Rabeler B., Zheng H., Raichlin E., Patel K.P. (2016). Exercise Training Attenuates Upregulation of p47(phox) and p67(phox) in Hearts of Diabetic Rats. Oxid. Med. Cell Longev..

[24] Guggilam A., Haque M., Kerut E.K., McIlwain E., Lucchesi P., Seghal I., Francis J. (2007). TNF-alpha blockade decreases oxidative stress in the paraventricular nucleus and attenuates sympathoexcitation in heart failure rats. Am. J. Physiol. Heart Circ. Physiol..

[25] Kerth S., Petry A., Kracun D., Morel O., Schini-Kerth V.B., Goerlach A. (2022). Sympathetic nervous system hyperactivity-induced oxidative stress promoting endothelial dysfunction is dependent on the NADPH oxidases/SGLT2 crosstalk: Potential role in cardiogenic shock. Eur. Hear. J..

[26] Patel K.P., Zhang K., Carmines P.K. (2000). Norepinephrine turnover in peripheral tissues of rats with heart failure. Am. J. Physiol. Regul. Integr. Comp. Physiol..

[27] Zheng H., Liu X., Katsurada K., Patel K.P. (2019). Renal denervation improves sodium excretion in rats with chronic heart failure: Effects on expression of renal ENaC and AQP2. Am. J. Physiol. Heart Circ. Physiol..

[28] Zheng H., Katsurada K., Liu X., Knuepfer M.M., Patel K.P. (2018). Specific Afferent Renal Denervation Prevents Reduction in Neuronal Nitric Oxide Synthase Within the Paraventricular Nucleus in Rats With Chronic Heart Failure. Hypertension.

[29] Su L.J., Zhang J.H., Gomez H., Murugan R., Hong X., Xu D., Jiang F., Peng Z.Y. (2019). Reactive Oxygen Species-Induced Lipid Peroxidation in Apoptosis, Autophagy, and Ferroptosis. Oxid. Med. Cell Longev..

[30] Keith M., Geranmayegan A., Sole M.J., Kurian R., Robinson A., Omran A.S., Jeejeebhoy K.N. (1998). Increased oxidative stress in patients with congestive heart failure. J. Am. Coll. Cardiol..

[31] Hermann M., Flammer A., Luscher T.F. (2006). Nitric oxide in hypertension. J. Clin. Hypertens..

[32] Daenen K., Andries A., Mekahli D., Van Schepdael A., Jouret F., Bammens B. (2019). Oxidative stress in chronic kidney disease. Pediatr. Nephrol..

[33] Irazabal M.V., Torres V.E. (2020). Reactive Oxygen Species and Redox Signaling in Chronic Kidney Disease. Cells.

[34] Kishi S., Nagasu H., Kidokoro K., Kashihara N. (2024). Oxidative stress and the role of redox signalling in chronic kidney disease. Nat. Rev. Nephrol..

[35] Gonzalez-Vicente A., Hong N., Garvin J.L. (2019). Effects of reactive oxygen species on renal tubular transport. Am. J. Physiol. Renal Physiol..

[36] Wang X.L., Li L., Meng X. (2024). Interplay between the Redox System and Renal Tubular Transport. Antioxidants.

[37] Gonzalez-Vicente A., Garvin J.L. (2017). Effects of Reactive Oxygen Species on Tubular Transport along the Nephron. Antioxidants.

[38] Hou X., Xiao H., Zhang Y., Zeng X., Huang M., Chen X., Birnbaumer L., Liao Y. (2018). Transient receptor potential channel 6 knockdown prevents apoptosis of renal tubular epithelial cells upon oxidative stress via autophagy activation. Cell Death Dis..

[39] Watanabe H., Miyamoto Y., Honda D., Tanaka H., Wu Q., Endo M., Noguchi T., Kadowaki D., Ishima Y., Kotani S. (2013). p-Cresyl sulfate causes renal tubular cell damage by inducing oxidative stress by activation of NADPH oxidase. Kidney Int..

[40] Thomas K., Zondler L., Ludwig N., Kardell M., Luneburg C., Henke K., Mersmann S., Margraf A., Spieker T., Tekath T. (2022). Glutamine prevents acute kidney injury by modulating oxidative stress and apoptosis in tubular epithelial cells. J. Clin. Investig..

[41] Sun Y., Ge X., Li X., He J., Wei X., Du J., Sun J., Li X., Xun Z., Liu W. (2020). High-fat diet promotes renal injury by inducing oxidative stress and mitochondrial dysfunction. Cell Death Dis..

[42] Fortuno A., Beloqui O., San Jose G., Moreno M.U., Zalba G., Diez J. (2005). Increased phagocytic nicotinamide adenine dinucleotide phosphate oxidase-dependent superoxide production in patients with early chronic kidney disease. Kidney Int. Suppl..

[43] Castilla P., Davalos A., Teruel J.L., Cerrato F., Fernandez-Lucas M., Merino J.L., Sanchez-Martin C.C., Ortuno J., Lasuncion M.A. (2008). Comparative effects of dietary supplementation with red grape juice and vitamin E on production of superoxide by circulating neutrophil NADPH oxidase in hemodialysis patients. Am. J. Clin. Nutr..

[44] Hoshino Y., Sonoda H., Nishimura R., Mori K., Ishibashi K., Ikeda M. (2019). Involvement of the NADPH oxidase 2 pathway in renal oxidative stress in *Aqp11*^−/−^ mice. Biochem. Biophys. Rep..

[45] Vaziri N.D., Dicus M., Ho N.D., Boroujerdi-Rad L., Sindhu R.K. (2003). Oxidative stress and dysregulation of superoxide dismutase and NADPH oxidase in renal insufficiency. Kidney Int..

[46] Matsuyama Y., Terawaki H., Terada T., Era S. (2009). Albumin thiol oxidation and serum protein carbonyl formation are progressively enhanced with advancing stages of chronic kidney disease. Clin. Exp. Nephrol..

[47] Szlagor M., Dybiec J., Mlynarska E., Rysz J., Franczyk B. (2023). Chronic Kidney Disease as a Comorbidity in Heart Failure. Int. J. Mol. Sci..

[48] Tedeschi A., Agostoni P., Pezzuto B., Corra U., Scrutinio D., La Gioia R., Raimondo R., Passantino A., Piepoli M.F. (2020). Role of comorbidities in heart failure prognosis Part 2: Chronic kidney disease, elevated serum uric acid. Eur. J. Prev. Cardiol..

[49] Scholten M., Davidge J., Agvall B., Halling A. (2024). Comorbidities in heart failure patients that predict cardiovascular readmissions within 100 days-An observational study. PLoS ONE.

[50] Jankowski J., Floege J., Fliser D., Bohm M., Marx N. (2021). Cardiovascular Disease in Chronic Kidney Disease: Pathophysiological Insights and Therapeutic Options. Circulation.

[51] Zoccali C., Mallamaci F., Adamczak M., de Oliveira R.B., Massy Z.A., Sarafidis P., Agarwal R., Mark P.B., Kotanko P., Ferro C.J. (2023). Cardiovascular complications in chronic kidney disease: A review from the European Renal and Cardiovascular Medicine Working Group of the European Renal Association. Cardiovasc. Res..

[52] Zoccali C., Mark P.B., Sarafidis P., Agarwal R., Adamczak M., Bueno de Oliveira R., Massy Z.A., Kotanko P., Ferro C.J., Wanner C. (2023). Diagnosis of cardiovascular disease in patients with chronic kidney disease. Nat. Rev. Nephrol..

[53] Griendling K.K., Camargo L.L., Rios F.J., Alves-Lopes R., Montezano A.C., Touyz R.M. (2021). Oxidative Stress and Hypertension. Circ. Res..

[54] Chung H.S., Wang S.B., Venkatraman V., Murray C.I., Van Eyk J.E. (2013). Cysteine oxidative posttranslational modifications: Emerging regulation in the cardiovascular system. Circ. Res..

[55] Sardesai V.M. (1995). Role of antioxidants in health maintenance. Nutr. Clin. Pract..

[56] Kurutas E.B. (2016). The importance of antioxidants which play the role in cellular response against oxidative/nitrosative stress: Current state. Nutr. J..

[57] Halliwell B. (1996). Antioxidants in human health and disease. Annu. Rev. Nutr..

[58] Nuttall S.L., Kendall M.J., Martin U. (1999). Antioxidant therapy for the prevention of cardiovascular disease. QJM Int. J. Med..

[59] Hoffman R.M., Garewal H.S. (1995). Antioxidants and the prevention of coronary heart disease. Arch. Intern. Med..

[60] Dennis J.M., Witting P.K. (2017). Protective Role for Antioxidants in Acute Kidney Disease. Nutrients.

[61] Nair S., Wilding J.P. (2010). Sodium glucose cotransporter 2 inhibitors as a new treatment for diabetes mellitus. J. Clin. Endocrinol. Metab..

[62] Hsia D.S., Grove O., Cefalu W.T. (2017). An update on sodium-glucose co-transporter-2 inhibitors for the treatment of diabetes mellitus. Curr. Opin. Endocrinol. Diabetes Obes..

[63] Kashiwagi A., Maegawa H. (2017). Metabolic and hemodynamic effects of sodium-dependent glucose cotransporter 2 inhibitors on cardio-renal protection in the treatment of patients with type 2 diabetes mellitus. J. Diabetes Investig..

[64] Talha K.M., Anker S.D., Butler J. (2023). SGLT-2 Inhibitors in Heart Failure: A Review of Current Evidence. Int. J. Heart Fail..

[65] Heerspink H.J., Perkins B.A., Fitchett D.H., Husain M., Cherney D.Z. (2016). Sodium Glucose Cotransporter 2 Inhibitors in the Treatment of Diabetes Mellitus: Cardiovascular and Kidney Effects, Potential Mechanisms, and Clinical Applications. Circulation.

[66] Girardi A.C.C., Polidoro J.Z., Castro P.C., Pio-Abreu A., Noronha I.L., Drager L.F. (2024). Mechanisms of heart failure and chronic kidney disease protection by SGLT2 inhibitors in nondiabetic conditions. Am. J. Physiol. Cell Physiol..

[67] Gao Y.M., Feng S.T., Wen Y., Tang T.T., Wang B., Liu B.C. (2022). Cardiorenal protection of SGLT2 inhibitors-Perspectives from metabolic reprogramming. EBioMedicine.

[68] Cowie M.R., Fisher M. (2020). SGLT2 inhibitors: Mechanisms of cardiovascular benefit beyond glycaemic control. Nat. Rev. Cardiol..

[69] Ferrannini E., Muscelli E., Frascerra S., Baldi S., Mari A., Heise T., Broedl U.C., Woerle H.J. (2014). Metabolic response to sodium-glucose cotransporter 2 inhibition in type 2 diabetic patients. J. Clin. Investig..

[70] Tsai K.F., Chen Y.L., Chiou T.T., Chu T.H., Li L.C., Ng H.Y., Lee W.C., Lee C.T. (2021). Emergence of SGLT2 Inhibitors as Powerful Antioxidants in Human Diseases. Antioxidants.

[71] Mulder S., Hammarstedt A., Nagaraj S.B., Nair V., Ju W., Hedberg J., Greasley P.J., Eriksson J.W., Oscarsson J., Heerspink H.J.L. (2020). A metabolomics-based molecular pathway analysis of how the sodium-glucose co-transporter-2 inhibitor dapagliflozin may slow kidney function decline in patients with diabetes. Diabetes Obes. Metab..

[72] Kawahito S., Kitahata H., Oshita S. (2009). Problems associated with glucose toxicity: Role of hyperglycemia-induced oxidative stress. World J. Gastroenterol..

[73] Giri B., Dey S., Das T., Sarkar M., Banerjee J., Dash S.K. (2018). Chronic hyperglycemia mediated physiological alteration and metabolic distortion leads to organ dysfunction, infection, cancer progression and other pathophysiological consequences: An update on glucose toxicity. Biomed. Pharmacother..

[74] Morales J., Handelsman Y. (2023). Cardiovascular Outcomes in Patients With Diabetes and Kidney Disease: JACC Review Topic of the Week. J. Am. Coll. Cardiol..

[75] Palsson R., Patel U.D. (2014). Cardiovascular complications of diabetic kidney disease. Adv. Chronic Kidney Dis..

[76] Chen L.H., Leung P.S. (2013). Inhibition of the sodium glucose co-transporter-2: Its beneficial action and potential combination therapy for type 2 diabetes mellitus. Diabetes Obes. Metab..

[77] Fonseca-Correa J.I., Correa-Rotter R. (2021). Sodium-Glucose Cotransporter 2 Inhibitors Mechanisms of Action: A Review. Front Med..

[78] Xu B., Li S., Kang B., Zhou J. (2022). The current role of sodium-glucose cotransporter 2 inhibitors in type 2 diabetes mellitus management. Cardiovasc. Diabetol..

[79] Teo Y.H., Teo Y.N., Syn N.L., Kow C.S., Yoong C.S.Y., Tan B.Y.Q., Yeo T.C., Lee C.H., Lin W., Sia C.H. (2021). Effects of Sodium/Glucose Cotransporter 2 (SGLT2) Inhibitors on Cardiovascular and Metabolic Outcomes in Patients Without Diabetes Mellitus: A Systematic Review and Meta-Analysis of Randomized-Controlled Trials. J. Am. Heart Assoc..

[80] Hasan I., Rashid T., Jaikaransingh V., Heilig C., Abdel-Rahman E.M., Awad A.S. (2024). SGLT2 inhibitors: Beyond glycemic control. J. Clin. Transl. Endocrinol..

[81] Nakagawa Y., Kuwahara K. (2020). Sodium-Glucose Cotransporter-2 inhibitors are potential therapeutic agents for treatment of non-diabetic heart failure patients. J. Cardiol..

[82] Kwon K. (2024). Newer Options for SGLT2 Inhibitors in the United States. Kidney News.

[83] Shoar S., Shah A.A., Ikram W., Farooq N., Udoh A., Tabibzadeh E., Khavandi S., Khavandi S. (2021). Cardiovascular benefits of SGLT2 inhibitors in patients with heart failure: A meta-analysis of small and large randomized controlled trials. Am. J. Cardiovasc. Dis..

[84] Kubota Y., Shimizu W. (2022). Clinical Benefits of Sodium-Glucose Cotransporter 2 Inhibitors and the Mechanisms Underlying Their Cardiovascular Effects. JACC Asia.

[85] Usman M.S., Siddiqi T.J., Anker S.D., Bakris G.L., Bhatt D.L., Filippatos G., Fonarow G.C., Greene S.J., Januzzi J.L., Khan M.S. (2023). Effect of SGLT2 Inhibitors on Cardiovascular Outcomes Across Various Patient Populations. J. Am. Coll. Cardiol..

[86] Mavrakanas T.A., Tsoukas M.A., Brophy J.M., Sharma A., Gariani K. (2023). SGLT-2 inhibitors improve cardiovascular and renal outcomes in patients with CKD: A systematic review and meta-analysis. Sci. Rep..

[87] Siddiqui R., Obi Y., Dossabhoy N.R., Shafi T. (2024). Is There a Role for SGLT2 Inhibitors in Patients with End-Stage Kidney Disease?. Curr. Hypertens. Rep..

[88] Wright E.M. (2021). SGLT2 Inhibitors: Physiology and Pharmacology. Kidney360.

[89] Scheen A.J. (2015). Pharmacokinetics, Pharmacodynamics and Clinical Use of SGLT2 Inhibitors in Patients with Type 2 Diabetes Mellitus and Chronic Kidney Disease. Clin. Pharmacokinet..

[90] Filippas-Ntekouan S., Tsimihodimos V., Filippatos T., Dimitriou T., Elisaf M. (2018). SGLT-2 inhibitors: Pharmacokinetics characteristics and effects on lipids. Expert. Opin. Drug Metab. Toxicol..

[91] Padda I.S., Mahtani A.U., Parmar M. (2025). Sodium-Glucose Transport Protein 2 (SGLT2) Inhibitors.

[92] Yurista S.R., Sillje H.H.W., Oberdorf-Maass S.U., Schouten E.M., Pavez Giani M.G., Hillebrands J.L., van Goor H., van Veldhuisen D.J., de Boer R.A., Westenbrink B.D. (2019). Sodium-glucose co-transporter 2 inhibition with empagliflozin improves cardiac function in non-diabetic rats with left ventricular dysfunction after myocardial infarction. Eur. J. Heart Fail..

[93] Cipriano A., Viviano M., Feoli A., Milite C., Sarno G., Castellano S., Sbardella G. (2023). NADPH Oxidases: From Molecular Mechanisms to Current Inhibitors. J. Med. Chem..

[94] Kolijn D., Pabel S., Tian Y., Lodi M., Herwig M., Carrizzo A., Zhazykbayeva S., Kovacs A., Fulop G.A., Falcao-Pires I. (2021). Empagliflozin improves endothelial and cardiomyocyte function in human heart failure with preserved ejection fraction via reduced pro-inflammatory-oxidative pathways and protein kinase Galpha oxidation. Cardiovasc. Res..

[95] Santos-Gallego C.G., Requena-Ibanez J.A., San Antonio R., Garcia-Ropero A., Ishikawa K., Watanabe S., Picatoste B., Vargas-Delgado A.P., Flores-Umanzor E.J., Sanz J. (2021). Empagliflozin Ameliorates Diastolic Dysfunction and Left Ventricular Fibrosis/Stiffness in Nondiabetic Heart Failure: A Multimodality Study. JACC Cardiovasc. Imag..

[96] Li C., Zhang J., Xue M., Li X., Han F., Liu X., Xu L., Lu Y., Cheng Y., Li T. (2019). SGLT2 inhibition with empagliflozin attenuates myocardial oxidative stress and fibrosis in diabetic mice heart. Cardiovasc. Diabetol..

[97] Oelze M., Kroller-Schon S., Welschof P., Jansen T., Hausding M., Mikhed Y., Stamm P., Mader M., Zinssius E., Agdauletova S. (2014). The sodium-glucose co-transporter 2 inhibitor empagliflozin improves diabetes-induced vascular dysfunction in the streptozotocin diabetes rat model by interfering with oxidative stress and glucotoxicity. PLoS ONE.

[98] Xing Y.J., Liu B.H., Wan S.J., Cheng Y., Zhou S.M., Sun Y., Yao X.M., Hua Q., Meng X.J., Cheng J.H. (2021). A SGLT2 Inhibitor Dapagliflozin Alleviates Diabetic Cardiomyopathy by Suppressing High Glucose-Induced Oxidative Stress in vivo and in vitro. Front. Pharmacol..

[99] Uthman L., Homayr A., Juni R.P., Spin E.L., Kerindongo R., Boomsma M., Hollmann M.W., Preckel B., Koolwijk P., van Hinsbergh V.W.M. (2019). Empagliflozin and Dapagliflozin Reduce ROS Generation and Restore NO Bioavailability in Tumor Necrosis Factor alpha-Stimulated Human Coronary Arterial Endothelial Cells. Cell Physiol. Biochem..

[100] Zhou Y., Tai S., Zhang N., Fu L., Wang Y. (2023). Dapagliflozin prevents oxidative stress-induced endothelial dysfunction via sirtuin 1 activation. Biomed. Pharmacother..

[101] Liu D.D., Liu X.L., Zheng T.F., Li X., Zhao Y.C., Pan J.C., Yuan C., Wang Q.Q., Zhang M. (2024). Dapagliflozin alleviates right heart failure by promoting collagen degradation by reducing ROS levels. Eur. J. Pharmacol..

[102] Peng Y., Guo M., Luo M., Lv D., Liao K., Luo S., Zhang B. (2024). Dapagliflozin ameliorates myocardial infarction injury through AMPKalpha-dependent regulation of oxidative stress and apoptosis. Heliyon.

[103] Ma H.X., Wu K., Dong F.H., Cai B.K., Wu D., Lu H.Y. (2024). Effects of Empagliflozin and Dapagliflozin in alleviating cardiac fibrosis through SIRT6-mediated oxidative stress reduction. Sci. Rep..

[104] Wan F., He X., Xie W. (2024). Canagliflozin Inhibits Palmitic Acid-Induced Vascular Cell Aging In Vitro through ROS/ERK and Ferroptosis Pathways. Antioxidants.

[105] Ionica L.N., Buriman D.G., Linta A.V., Sosdean R., Lascu A., Streian C.G., Feier H.B., Petrescu L., Mozos I.M., Sturza A. (2024). Empagliflozin and dapagliflozin decreased atrial monoamine oxidase expression and alleviated oxidative stress in overweight non-diabetic cardiac patients. Mol. Cell Biochem..

[106] Kaludercic N., Carpi A., Menabo R., Di Lisa F., Paolocci N. (2011). Monoamine oxidases (MAO) in the pathogenesis of heart failure and ischemia/reperfusion injury. Biochim. Biophys. Acta.

[107] Maggiorani D., Manzella N., Edmondson D.E., Mattevi A., Parini A., Binda C., Mialet-Perez J. (2017). Monoamine Oxidases, Oxidative Stress, and Altered Mitochondrial Dynamics in Cardiac Ageing. Oxid. Med. Cell Longev..

[108] Sun P., Wang Y., Ding Y., Luo J., Zhong J., Xu N., Zhang Y., Xie W. (2021). Canagliflozin attenuates lipotoxicity in cardiomyocytes and protects diabetic mouse hearts by inhibiting the mTOR/HIF-1alpha pathway. iScience.

[109] Du S., Shi H., Xiong L., Wang P., Shi Y. (2022). Canagliflozin mitigates ferroptosis and improves myocardial oxidative stress in mice with diabetic cardiomyopathy. Front. Endocrinol..

[110] Kondo H., Akoumianakis I., Badi I., Akawi N., Kotanidis C.P., Polkinghorne M., Stadiotti I., Sommariva E., Antonopoulos A.S., Carena M.C. (2021). Effects of canagliflozin on human myocardial redox signalling: Clinical implications. Eur. Heart J..

[111] Nishinarita R., Niwano S., Niwano H., Nakamura H., Saito D., Sato T., Matsuura G., Arakawa Y., Kobayashi S., Shirakawa Y. (2021). Canagliflozin Suppresses Atrial Remodeling in a Canine Atrial Fibrillation Model. J. Am. Heart Assoc..

[112] Sabe S.A., Xu C.M., Sabra M., Harris D.D., Malhotra A., Aboulgheit A., Stanley M., Abid M.R., Sellke F.W. (2023). Canagliflozin Improves Myocardial Perfusion, Fibrosis, and Function in a Swine Model of Chronic Myocardial Ischemia. J. Am. Heart Assoc..

[113] The E.-K.C.G., Herrington W.G., Staplin N., Wanner C., Green J.B., Hauske S.J., Emberson J.R., Preiss D., Judge P., Mayne K.J. (2023). Empagliflozin in Patients with Chronic Kidney Disease. N. Engl. J. Med..

[114] Nuffield Department of Population Health Renal Studies Group, SGLT2 inhibitor Meta-Analysis Cardio-Renal Trialists’ Consortium (2022). Impact of diabetes on the effects of sodium glucose co-transporter-2 inhibitors on kidney outcomes: Collaborative meta-analysis of large placebo-controlled trials. Lancet.

[115] Group E.-K.C. (2024). Effects of empagliflozin on progression of chronic kidney disease: A prespecified secondary analysis from the empa-kidney trial. Lancet Diabetes Endocrinol..

[116] Lee W.C., Chau Y.Y., Ng H.Y., Chen C.H., Wang P.W., Liou C.W., Lin T.K., Chen J.B. (2019). Empagliflozin Protects HK-2 Cells from High Glucose-Mediated Injuries via a Mitochondrial Mechanism. Cells.

[117] Lu Q., Yang L., Xiao J.J., Liu Q., Ni L., Hu J.W., Yu H., Wu X., Zhang B.F. (2023). Empagliflozin attenuates the renal tubular ferroptosis in diabetic kidney disease through AMPK/NRF2 pathway. Free Radic. Biol. Med..

[118] Das N.A., Carpenter A.J., Belenchia A., Aroor A.R., Noda M., Siebenlist U., Chandrasekar B., DeMarco V.G. (2020). Empagliflozin reduces high glucose-induced oxidative stress and miR-21-dependent TRAF3IP2 induction and RECK suppression, and inhibits human renal proximal tubular epithelial cell migration and epithelial-to-mesenchymal transition. Cell Signal.

[119] Ashrafi Jigheh Z., Ghorbani Haghjo A., Argani H., Roshangar L., Rashtchizadeh N., Sanajou D., Nazari Soltan Ahmad S., Rashedi J., Dastmalchi S., Mesgari Abbasi M. (2019). Empagliflozin alleviates renal inflammation and oxidative stress in streptozotocin-induced diabetic rats partly by repressing HMGB1-TLR4 receptor axis. Iran. J. Basic. Med. Sci..

[120] Malinska H., Huttl M., Markova I., Miklankova D., Hojna S., Papousek F., Silhavy J., Mlejnek P., Zicha J., Hrdlicka J. (2022). Beneficial Effects of Empagliflozin Are Mediated by Reduced Renal Inflammation and Oxidative Stress in Spontaneously Hypertensive Rats Expressing Human C-Reactive Protein. Biomedicines.

[121] Mihaljevic V., Zjalic M., Kizivat T., Omanovic Kolaric T., Smolic M., Rodak E., Covic M., Kuna L., Smolic R., Vcev A. (2022). Molecular Mechanisms Linking Empagliflozin to Renal Protection in the LLC-PK1 Model of Diabetic Nephropathy. Biomedicines.

[122] Jin J., Jin L., Luo K., Lim S.W., Chung B.H., Yang C.W. (2017). Effect of Empagliflozin on Tacrolimus-Induced Pancreas Islet Dysfunction and Renal Injury. Am. J. Transplant..

[123] Zeng X.C., Tian Y., Liang X.M., Wu X.B., Yao C.M., Chen X.M. (2024). SGLT2i relieve proteinuria in diabetic nephropathy patients potentially by inhibiting renal oxidative stress rather than through AGEs pathway. Diabetol. Metab. Syndr..

[124] Ding T., Song M., Wang S., Huang C., Pan T. (2025). Dapagliflozin has protective effects on palmitate-induced renal tubular epithelial cells by enhancing mitochondrial function and reducing oxidative stress. J. Diabetes Its Complicat..

[125] Jaikumkao K., Pongchaidecha A., Chueakula N., Thongnak L., Wanchai K., Chatsudthipong V., Chattipakorn N., Lungkaphin A. (2018). Renal outcomes with sodium glucose cotransporter 2 (SGLT2) inhibitor, dapagliflozin, in obese insulin-resistant model. Biochim. Biophys. Acta Mol. Basis Dis..

[126] Swe M.T., Thongnak L., Jaikumkao K., Pongchaidecha A., Chatsudthipong V., Lungkaphin A. (2020). Dapagliflozin attenuates renal gluconeogenic enzyme expression in obese rats. J. Endocrinol..

[127] Zaibi N., Li P., Xu S.Z. (2021). Protective effects of dapagliflozin against oxidative stress-induced cell injury in human proximal tubular cells. PLoS ONE.

[128] Zeng J., Huang H., Zhang Y., Lv X., Cheng J., Zou S.J., Han Y., Wang S., Gong L., Peng Z. (2023). Dapagliflozin alleviates renal fibrosis in a mouse model of adenine-induced renal injury by inhibiting TGF-beta1/MAPK mediated mitochondrial damage. Front. Pharmacol..

[129] Cinakova A., Vavrincova-Yaghi D., Krenek P., Klimas J., Kralova E. (2025). Combination of dapagliflozin and pioglitazone lacks superiority against monotherapy in streptozotocin-induced nephropathy. Sci. Rep..

[130] Terami N., Ogawa D., Tachibana H., Hatanaka T., Wada J., Nakatsuka A., Eguchi J., Horiguchi C.S., Nishii N., Yamada H. (2014). Long-term treatment with the sodium glucose cotransporter 2 inhibitor, dapagliflozin, ameliorates glucose homeostasis and diabetic nephropathy in db/db mice. PLoS ONE.

[131] Urbanek K., Cappetta D., Bellocchio G., Coppola M.A., Imbrici P., Telesca M., Donniacuo M., Riemma M.A., Mele E., Cianflone E. (2023). Dapagliflozin protects the kidney in a non-diabetic model of cardiorenal syndrome. Pharmacol. Res..

[132] Hasan R., Lasker S., Hasan A., Zerin F., Zamila M., Parvez F., Rahman M.M., Khan F., Subhan N., Alam M.A. (2020). Canagliflozin ameliorates renal oxidative stress and inflammation by stimulating AMPK-Akt-eNOS pathway in the isoprenaline-induced oxidative stress model. Sci. Rep..

[133] Kimura Y., Kuno A., Tanno M., Sato T., Ohno K., Shibata S., Nakata K., Sugawara H., Abe K., Igaki Y. (2019). Canagliflozin, a sodium-glucose cotransporter 2 inhibitor, normalizes renal susceptibility to type 1 cardiorenal syndrome through reduction of renal oxidative stress in diabetic rats. J. Diabetes Investig..

[134] Ali B.H., Al Salam S., Al Suleimani Y., Al Za’abi M., Abdelrahman A.M., Ashique M., Manoj P., Adham S.A., Hartmann C., Schupp N. (2019). Effects of the SGLT-2 Inhibitor Canagliflozin on Adenine-Induced Chronic Kidney Disease in Rats. Cell Physiol. Biochem..

[135] Liu H., Chen W., Wan S., Chen Y., Fu M., Wang Z., Xiong F., Zhang Y. (2023). Canagliflozin ameliorates high glucose-induced apoptosis in NRK-52E cells via inhibiting oxidative stress and activating AMPK/mTOR-mediated autophagy. Mol. Biol. Rep..

